# Hop Flower Supercritical Carbon Dioxide Extracts Coupled with Carriers with Solubilizing Properties—Antioxidant Activity and Neuroprotective Potential

**DOI:** 10.3390/antiox12091722

**Published:** 2023-09-05

**Authors:** Anna Stasiłowicz-Krzemień, Judyta Cielecka-Piontek

**Affiliations:** 1 Department of Pharmacognosy and Biomaterials, Faculty of Pharmacy, Poznan University of Medical Sciences, Rokietnicka 3, 60-806 Poznan, Poland; 2 Department of Pharmacology and Phytochemistry, Institute of Natural Fibres and Medicinal Plants, Wojska Polskiego 71b, 60-630 Poznan, Poland

**Keywords:** neuroprotection, hop strobile, inhibition of enzymes, xanthohumol

## Abstract

*Lupuli flos* shows many biological activities like antioxidant potential, extended by a targeted effect on selected enzymes, the expression of which is characteristic for neurodegenerative changes within the nervous system. *Lupuli flos* extracts (LFE) were prepared by supercritical carbon dioxide (scCO_2_) extraction with various pressure and temperature parameters. The antioxidant, chelating activity, and inhibition of acetylcholinesterase (AChE), butyrylcholinesterase (BChE), and tyrosinase by extracts were studied. The extracts containing ethanol were used as references. The most beneficial neuroprotective effects were shown by the extract obtained under 5000 PSI and 50 °C. The neuroprotective effect of active compounds is limited by poor solubility; therefore, carriers with solubilizing properties were used for scCO_2_ extracts, combined with post-scCO_2_ ethanol extract. Hydroxypropyl-β-cyclodextrin (HP-β-CD) in combination with magnesium aluminometasilicate (Neusilin US2) in the ratio 1:0.5 improved dissolution profiles to the greatest extent, while the apparent permeability coefficients of these compounds determined using the parallel artificial membrane permeability assay in the gastrointestinal (PAMPA GIT) model were increased the most by only HP-β-CD.

## 1. Introduction

Neurodegeneration is a major problem that affects millions of people worldwide. It is a progressive loss of function and structure of neurons, leading to a decline in cognitive and motor function. Neurodegenerative diseases can be categorized based on the main clinical features such as motor neuron disease, dementia, or parkinsonism, the anatomic distribution of neurodegeneration such as extrapyramidal disorders, frontotemporal degenerations, or spinocerebellar degenerations, or the primary molecular abnormality [1].

Despite extensive research efforts, there are currently no effective treatments to cure or halt the progression of these disorders [2]. Neurodegenerative diseases are often accompanied by characteristic excitability and insomnia. The pharmacological solutions have numerous side effects, including the development of drug tolerance. In the case of raw plant materials, it is possible for the ingredients to act holistically within the central nervous system (CNS), including the possibility of inducing safe sedation while neutralizing free radicals generated in neurodegeneration processes, inhibiting the activity of enzymes whose expression causes neurodegenerative changes or even anti-inflammatory effects. One of the plant materials that has a holistic effect on the CNS is hop strobile. *Lupuli flos* has been used since ancient times as a remedy for many ailments [3]. Hop strobiles have been studied for many conditions and symptoms in both their treatment and prevention. As this plant material is favored in many industries (the cosmetic industry, food industry, beverage industry, and pharmaceutical industry), and often used by patients, the preparations on the market should be as efficient as possible in specific directions. It is the female inflorescence of the hop plant (*Humulus lupulus* L., family *Cannabaceae*) that is known for its anti-stress potential, promoting and improving sleep and easing anxiety [4]. What is more, this plant material exerts antioxidant properties as it scavenges free radicals, reduces lipid peroxidation, and upregulates the activity of antioxidant enzymes: superoxide dismutase and glutathione peroxidase [5]. Hop strobile provides neuroprotection from oxidative stress and prevents neuronal damage and neuron death; it also takes part in inhibiting the development of neurodegenerative diseases [6]. Hop strobile contains a variety of bioactive compounds, including prenylflavonoids such as xanthohumol, terpenes such as humulene and caryophyllene, flavonoids, and tannins, as well as bitter acids: α-acids and β-acids [7].

Secondary metabolites of hop strobile might be extracted with various solvents and methods. One of the extraction methods is supercritical carbon dioxide (scCO_2_) extraction. The lower temperatures required for scCO_2_ extraction can help preserve the delicate chemical structures of heat-sensitive compounds, which may be destroyed by higher temperature methods such as terpenes or derivatives of prenylflavonoids.

Compounds of hop strobile which act within the CNS (xanthohumol [8], for example, 6-prenylnaringenin [9], 8-prenylnaringenin [10,11], bitter acids [12], and flavonoids such as quercetin and kaempferol [13,14,15]) are lipophilic. This property allows them to penetrate the blood–brain barrier. However, as neuroprotective agents, the potential for their use is limited by their poor solubility in water, which limits their absorption at the stage of absorption in the digestive system. That is why there are articles present in the literature on improving the solubility and, consequently, increasing the bioavailability of valuable compounds derived from hop cones. For example, for the xanthohumol microemulsion delivery system based on biosurfactant sophorolipids [16], pluronic-based drug delivery systems [17], xanthohumol c/hydroxypropyl-β-cyclodextrin (HP-β-CD) complex [18], cocrystals [19], and the guar gum-pectin-based colon-targeted solid self-nanoemulsifying drug delivery system [20] were prepared.

Therefore, the aim of our research was to find the most suitable parameters of scCO_2_ extraction of *Lupuli flos*, optimizing its antioxidant and neuroprotective potential. Concurrently, our goal was to enhance the solubility and membrane permeability of the secondary metabolites present in *Lupuli flos* by using carriers with solubilizing properties—HP-β-CD and magnesium aluminometasilicate (Neusilin US2) and their impact on the biological activity of the extract.

## 2. Materials and Methods

### 2.1. Materials

Plant material, *Lupuli flos*, which is registered as an over-the-counter drug in Poland was purchased from Zakład Zielarski KAWON-HURT, Gostyń, Poland (serial number: 256.2022). HP-β-CD (molar substitution 0.8, Mw~1.460) was purchased from Sigma-Aldrich (Poznan, Poland). Neusilin US2 was kindly provided by Fuji Chemical Industry (Minato, Tokyo). Sigma-Aldrich (Poznan, Poland) provided lupulone and xanthohumol. Merck (Darmstadt, Germany) provided the trifluoroacetic acid and high-performance liquid chromatography (HPLC)-grade methanol. High-quality pure water was generated using a Direct-Q 3 UV purification system (Millipore, Molsheim, France; model Exil SA 67120). 5,5-dithio-bis-(2-nitrobenzoic acid), 2,2-Diphenyl-1-picrylhydrazyl (DPPH), iron (III) chloride hexahydrate, 2,2′-azino-bis(3-ethylbenzothiazoline-6-sulfonic acid), neocuproine, 2,4,6-Tri(2-pyridyl)-s-triazine, trolox, tyrosinase from mushroom, L-DOPA, acetylcholinesterase from *Electrophorus electricus* (AChE), butyrylcholinesterase from equine serum (BChE), acetylcholine iodide (ATCI), butyrylcholine iodide (BTCI), Trizma^®^ hydrochloride, and Trizma^®^ base were also purchased from Sigma-Aldrich (Schnelldorf, Germany). The chemicals sodium chloride, sodium dihydrogen phosphate, and sodium hydrogen phosphate were obtained from Avantor Performance Materials (Gliwice, Poland), while ammonium acetate and methanol were obtained from Chempur (Piekary Śląskie, Poland). Finally, cupric chloride dihydrate, acetic acid (99.5%), ethanol (96%), and sodium acetate trihydrate were provided by POCH (Gliwice, Poland).

### 2.2. Extraction Process

Dry raw hop strobiles were ground and 3.0 g of the material was placed in an extraction vessel. The dynamic scCO_2_ extraction process was performed under 2000, 3500, and 6000 PSI at 40 °C, and 50 °C with 150 mL of CO_2_ (Table 1). Next, the extracts were suspended in 70% ethanol. The post-scCO_2_ material was withdrawn from the extraction vessel, placed in a conical flask, and 90 mL of 70% ethanol was added. The post-scCO_2_ material was extracted at the same temperature as in scCO_2_ extraction for 60 min, in an ultrasonic bath, twice (each time using fresh extractant).

### 2.3. Chromatographic Analysis

**Figure 1:**
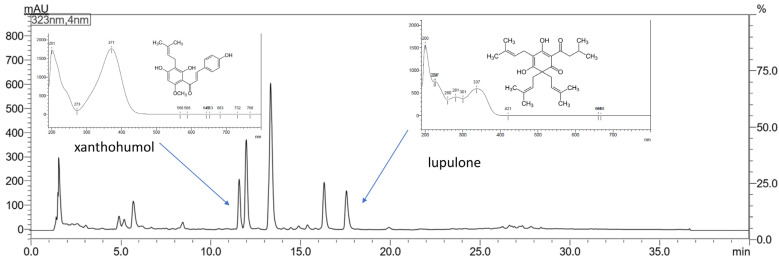
The chromatogram of xanthohumol and lupulone present in Hop strobile extracts.

The chromatographic analysis was performed using high-performance liquid chromatography with the diode array detector (HPLC-DAD) method (Shimadzu Corp., Kyoto, Japan). The determination was performed with a validated method using the stationary phase: column of ReproShell PFP (150 mm × 4.6 mm; 2.7 µm). The mobile phase was composed of 0.1% trifluoroacetic acid (A), and methanol (B) with a gradient elution of 0–5 min B: 50–60%, 5–15 min B: 60–70%, 15–20 B: 70%, 20–25 min B: 70–80%, 25–30 min B: 80–85%, 30–35 min B: 85%. The flow rate was set at 1.0 mL/min, and the column temperature was 40 °C. The injection volume was 10.0 µL, whilst the detection wavelength was set at 323 nm. The retention time of xanthohumol was approx. at 11.7 min, whilst for lupulone it was at 17.6 min (Figure 1). The results were obtained and processed by LabSolutions LC software (version 1.86 SP2, Shimadzu Corp., Kyoto, Japan).

### 2.4. Analysis of Total Phenolic Content

TPC was performed using the Folin–Ciocalteu method with minor modifications according to Sip. et al. [21]. The assay was performed on a 96-well plate, and the samples were measured colorimetrically. A total of 50.0 µL of extract/gallic acid was pipetted to the plate and 50.0 µL of Folin–Ciocalteu reagent and 100.0 µL of distilled water were also added and incubated in dark conditions, for 5 min, at 37 °C, while shaking. Subsequently, 100 µL of 20% Na_2_CO_3_ solution was added and the plate was later incubated under the same conditions for 30 min. Absorbance was measured at 750 nm against the blank sample (water instead of the extract/gallic acid) using a plate reader (Multiskan GO, Thermo Fisher Scientific, Waltham, MA, USA). Each sample was also analyzed for its own absorbance at 750 nm. The samples were studied in sextuplicate. A linear regression equation between the gallic acid concentration and absorbance was prepared, and the results were presented as mg of gallic acid equivalent (GAE) per 1 g of plant material.

### 2.5. Inhibition of Enzymes Influencing the Development of the Neurodegenerative Diseases

The potential of the extracts to inhibit enzymes involved in neurodegeneration development, AChE, BChE, and tyrosinase was studied. The ability to inhibit AChE and BChE was determined using a modified spectrometric assay developed by Ellman et al. [22]. This technique involves the use of synthetic substrates known as thiocholine esters. Thiocholine is liberated during the enzymatic reactions with 5,5′-dithio-bis-(2-nitrobenzoic) acid (DTNB), and the 3-carboxy-4-nitrothiolate anion (TNB anion) is formed. The procedural steps were executed on a 96-well plate [23]. Within each well, 60.0 μL of 0.05 M Tris-HCl buffer (pH of 8.0), 10.0 μL of the extract, and 30.0 μL of AChE/BChE solution at a concentration of 0.2 U/mL were pipetted to the wells. Subsequently, the plate was subjected to a 5 min incubation phase at 37 °C with continuous agitation. Following this, 30.0 μL of 1.5 mM ATCI/BTCI solution and 125.0 μL of 0.3 mM DTNB solution (5,5′-dithiobis-(2-nitrobenzoic acid) were added to the wells and the plate was once more subjected to a 20 min incubation under identical conditions. A blank for the test sample (the reaction mixture was stripped of the enzyme, and the volume of Tris-HCl buffer was elevated), the control sample (solvent was added instead of the test sample), and the blank for the control sample (the reaction mixture of the control sample was depleted of the enzyme (the volume of Tris-HCl buffer was raised)) were also prepared. The measurements were colorimetrically performed at 405 nm. The percentage of AChE and BChE inhibition by the samples was calculated using the equation provided:

(1) $$\mathrm{AChE/BChE}\;\mathrm{inhibition}\;(\%)=\frac{1-\left(A_{1}-A_{1b}\right)}{\left(A_{0}-A_{0b}\right)}\times 100\%$$

where:

*A*_1_—the absorbance of the test sample;

*A*_1*b*_—the absorbance of the blank of the test sample;

*A*_0_—the absorbance of control;

*A*_0*b*_—the absorbance of the blank of control.

From the obtained results, the IC_50_ value was calculated, which corresponds to the concentration of extract required to inhibit 50% of the enzyme activity.

The tyrosinase inhibition assay is based on measuring the reduction in color intensity of a solution due to the inhibition of enzyme activity [24]. The inhibitor used in this assay prevents L-DOPA from accessing the tyrosinase active site, thereby halting the reaction. To perform the assay, 75.0 μL of 0.1 M phosphate buffer with a pH of 6.8, 25.0 μL of the extract, and 50.0 μL of enzyme solution (192 U/mL) were added to the wells of a 96-well plate, which was then incubated while shaking at room temperature for 10 min. Afterward, 50 μL of 2.0 mM L-DOPA was added to the wells and incubated in the same conditions for an additional 20 min. A blank for the test sample (the enzyme was removed from the reaction mixture, and the volume of phosphate buffer was increased), the control sample (solvent was introduced instead of the test sample), and the blank for the control sample (the enzyme was eliminated from the reaction mixture of the control sample (the volume of phosphate buffer was heightened)) were also prepared. At a wavelength of 475 nm, the absorbance of the test samples was measured, facilitating the calculation of the extent of tyrosinase inhibition induced by the samples using the equation provided.

(2) $$\mathrm{Tyrosinase}\;\mathrm{inhibition}\;(\%)=\frac{1-\left(A_{1}-A_{1b}\right)}{\left(A_{0}-A_{0b}\right)}\times 100\%$$

where:

*A*_1_—the absorbance of the test sample;

*A*_1*b*_—the absorbance of the blank of the test sample;

*A*_0_—the absorbance of control;

*A*_0*b*_—the absorbance of the blank of control.

From the obtained results, the IC_50_ value was calculated, which corresponds to the concentration of extract required to inhibit 50% of the enzyme activity.

### 2.6. Studies of the Antioxidant Activity of Extracts

Four methods (DPPH, 2:2′-azino-bis(3-ethylbenzothiazoline-6-sulfonic acid) (ABTS), cupric reducing antioxidant capacity (CUPRAC), and ferric reducing antioxidant power (FRAP)) were used to measure the antioxidant activity. The extracts were screened at decreasing concentrations before each assay was conducted. Trolox antioxidant activity was also examined to determine its ability to inhibit radicals (DPPH and ABTS) or perform redox reactions (CUPRAC and FRAP). A linear equation was created to relate the trolox concentration to its scavenging percentage (DPPH and ABTS) or absorbance (CUPRAC and FRAP). The results were then calculated as mg trolox/g plant material using the standard Trolox curve equation, based on the antioxidant properties of the extracts in all four assays [25,26]. The antioxidant properties of pure carriers were also studied in all four assays.

The DPPH assay was performed on a 96-well plate, and all the samples were measured using spectrophotometry [27]. As the main reagent, a methanol solution of DPPH (0.2 mM) was used. To initiate the assay, 25.0 µL of the extract/trolox/system was mixed with 175.0 µL of the DPPH solution. The plate was then placed in a dark, room-temperature environment and shaken for 30 min. Absorbance readings were obtained at 517 nm using a plate reader (Multiskan GO, Thermo Fisher Scientific, Waltham, MA, USA). The absorbance (A) was also measured simultaneously for the blank (a mixture of DPPH solution and solvent) at 517 nm. Each sample was analyzed for its own absorbance at 517 nm. The inhibition of DPPH radicals by the extract/trolox/system was calculated using the following formula:

(3) $$\mathrm{DPPH}\;\mathrm{scavenging}\;\mathrm{activity}\;\left(\%\right)\;\frac{A_{o\;}-A_{i}}{A_{o}}\times 100\%$$

where *A_o_* is the absorbance of the control sample and *A_i_* is the absorbance of the test sample. Each measurement was repeated six times.

The ABTS assay [28] was also used to determine the ability to scavenge radicals. This method involves the production of green cation radicals from ABTS through the loss of electrons by nitrogen atoms caused by potassium persulfate. During the assay, an antioxidant converts the green ABTS radical into a colorless neutral form. To perform the assay, 200.0 μL of ABTS^•+^ solution and 10.0 μL of the extract/trolox/system were pipetted into 96-well plates and incubated for 10 min in the dark at room temperature while shaking [23]. The absorbance values were then measured at λ = 734 nm using a Multiskan GO plate reader (Thermo Fisher Scientific, Waltham, MA, USA). Control mixtures of solvent and ABTS were also tested, along with wells containing samples and water to measure their own absorbance at 734 nm. The inhibition of ABTS^•+^ by the extract/trolox/system was calculated using the following formula:

(4) $$\mathrm{ABTS}\;\mathrm{scavenging}\;\mathrm{activity}\;(\%)=\frac{A_{0}-A_{1}}{A_{0}}\times 100\%$$

where:

*A*_0_—the absorbance of the control;

*A*_1_—the absorbance of the sample.

The CUPRAC assay was used to determine the extracts and system reduction potential [29]. In this technique, the oxidant’s phenolic groups are transformed into quinones, and the neocuproine and copper (II) ion complex (bluish) is transformed into the neocuproine and copper (I) ion complex (yellow). For the study, 50.0 µL of the extract/trolox/system and 150.0 µL of the CUPRAC reagent were mixed in the plate and incubated in the dark and at room temperature with shaking for 30 min [23]. The control and extracts’ own absorbance were also evaluated simultaneously. After 30 min, the absorbance was measured at a wavelength of 450 nm (Multiskan GO, Thermo Fisher Scientific, Waltham, MA, USA).

The FRAP method was the final antioxidant technique used to determine the extracts and reducing properties, where the colorless Fe^3+^ ion is reduced to Fe^2+^ and forms a dark blue complex with 2,4,6-tris(2-pyridyl)-1,3,5-triazine (TPTZ) [23]. The technique involved adding 25.0 µL of the extract/trolox/system and 175.0 µL of the FRAP mixture (25 mL acetate buffer, 2.5 mL TPTZ solution, and 2.5 mL of FeCl_3_·6H_2_O solution) to the plate and incubating it for 30 min in dark conditions at 37 °C. The control and extracts’ absorbance were also examined. Subsequently, the absorbance was measured at a wavelength of λ = 593 nm. (Multiskan GO, Thermo Fisher Scientific, Waltham, MA, USA). The analysis was performed in six replicates.

### 2.7. Chelating Properties of Lupuli Flos Extracts

The Fe^2+^ chelation ability of the extracts was determined on a 96-well plate according to Studzińska-Sroka et al. [30]. To perform the assay, 200.0 µL of studied extracts, and 10.0 µL of iron (II) chloride aqueous solution were pipetted to the wells. Subsequently, the plate was shaken in dark conditions, at room temperature, for 10 min. Later 40.0 µL of ferrozine aqueous solution was added and the plate was incubated under the same conditions for another 10 min. The samples were studied in sextuplicate, and the absorbance was measured at λ = 562 nm. The control sample contained 200.0 µL of the solvent and 10.0 µL of iron (II) aqueous chloride solution. The blanks were prepared without the ferrozine replacing it with distilled water. The chelating capacity of iron (II) ions was calculated using the equation:

(5) $${\mathrm{Fe}}^{2+}\;\mathrm{chelating}\;\mathrm{activity}\;\left(\%\right)=1-\frac{A_{s\;}-A_{bs}}{A_{c\;}-A_{bc}}\times 100$$

where:

*A_s_* is the absorbance of the sample, *A_bs_* is the absorbance of the blank of the sample, *A_c_* is the absorbance of the control, and *A_bc_* is the absorbance of the blank of the control. From the obtained results, the IC_50_ value was calculated.

### 2.8. Preparation of the Systems

After identifying the scCO_2_ extract with the most promising neuroprotective properties, we proceeded to prepare a fresh batch of the extract using identical conditions as before. Subsequently, the remaining plant material underwent extraction using ethanol. Both the scCO_2_ extract and the ethanol fractions were combined to capture a wider range of secondary plant metabolites and enhance the overall biological activity. This strategy aimed to optimize the neuroprotective potential of the final extract, thus increasing its efficacy for potential therapeutic applications.

The fractions were studied with HPLC, and the process was repeatable. To fluid extracts, carriers of solubilizing properties: Neusilin US2, and HP-β-CD were added in 1:1.5 ratio (to overcome the adhesive properties of the extract, based on preliminary studies on uniformity and flowability) plant material:excipients to obtain proper properties of the powder (Table 2):

The mixture was frozen at −22 °C, and then freeze-dried with the use of a lyophilizer (Heto PowerDry PL3000) Freeze Dryer (Thermo Scientific, Waltham, MA, USA) for 72 h to remove all water from the systems; the automatic procedure programmed in the freeze dryer was used. The resulting formulation was grounded in a mortar. The systems were studied by the HPLC-DAD.

### 2.9. Apparent Solubility Study

The dissolution rate was determined in the paddle apparatus (Agilent Technologies, Santa Clara, CA, USA). The systems were placed into gelatin capsules and implemented into springs for floating prevention. The extract itself was excluded from the study as it was impossible to dry and powderize the extract without adding excipients. The test was carried out for 180 min in Fasted State Simulated Gastric Fluid (FaSSGF), Fasted State Simulated Intestinal Fluid (FaSSIF), and Fed State Simulated Intestinal Fluid (FeSSIF).

FaSSGF, FaSSIF, and FeSSIF media were prepared according to the instructions of the manufacturer (Biorelevant, London, UK). The vessels were filled with 500 mL of media at the temperature set at 37 ± 0.5 °C and the rotation speed of 100 rpm. At specific time intervals, 2.0 mL of the sample was taken out and immediately replaced with an equal amount of fresh medium at the same temperature. The samples were then passed through a filter with a pore size of 0.22 μm and analyzed using high-performance liquid chromatography (HPLC). The differences and similarities among the profiles were examined by the two-factor values, *f*_1_, and *f*_2_, introduced by Moore and Flanner [31]. This analysis was executed employing the subsequent set of equations.

(6) $$f_{1}=\frac{\sum_{j=1}^{n}\left|R_{j}-T_{j}\right|}{\sum_{j=1}^{n}R_{j}}$$

(7) $$f_{2}=50\times \log\left({\left(1+\left(\frac{1}{n}\right)\sum_{j=1}^{n}{\left|R_{j}-T_{j}\right|}^{2}\right)}^{-\frac{1}{2}}\times 100\right)$$

where *n* is the number of time points, *R_j_* is the percentage of the dissolved reference substance in the medium, *T_j_* is the percentage of the dissolved tested substance, and *j* is the time point. Similarity within dissolution profiles is indicated by an *f*_1_ value approaching zero or an *f*_2_ value nearing 100, falling within the range of 50 to 100 [32].

### 2.10. Permeability Study

The permeability of xanthohumol and lupulone through biological membrane simulating conditions in the gastrointestinal tract was measured using the parallel artificial membrane permeability assay (PAMPA) model. The study was conducted in the gastrointestinal (GIT) and blood–brain barrier (BBB) models. The model consists of two 96-well microfilter plates, the donor chamber, and the acceptor chamber. These chambers were separated by a 120 μm-thick microfilter disc coated with a 20% (*w*/*v*) dodecane solution of a lecithin mixture (Pion Inc., Billerica, MA, USA). The extract was diluted and systems were dissolved/suspended (and centrifuged if needed) in dimethyl sulfoxide (DMSO) and placed in the donor solutions, which were adjusted to pH 1.2 and 6.8 for GIT application and pH 7.4 for BBB. The BBB permeability was only studied for the extract, as CDs, and Neu do not leave the GIT. The plates were incubated at 37 °C for 3 h for the GIT and for 4 h for the BBB assay in a humidity-saturated atmosphere. After incubation, the plates were separated and the concentration of xanthohumol and lupulone was determined using the HPLC-DAD method. The permeability coefficient was calculated using the formulas:

(8) $$P_{app}=\frac{-ln\left(1-\frac{C_{A}}{C_{equilibrium}}\right)}{S\times \left(\frac{1}{V_{D}}+\frac{1}{V_{A}}\right)\times t}$$

(9) $$C_{equilibrium}=\frac{C_{D}\times V_{D}+C_{A}\times V_{A}}{V_{D}+V_{A}}$$

where: *V_D_*—donor volume, *V_A_*—acceptor volume, *C_equilibrium_*—equilibrium concentration $C_{equilibrium}=\frac{C_{D}\times V_{D}+C_{A}\times V_{A}}{V_{D}+V_{A}}$, *S*—membrane area, and *t*—incubation time (in seconds).

Substances with a *P_app_* in the GIT model below 0.1 × 10^−6^ cm/s are considered to have poor permeability, compounds with 0.1 × 10^−6^ cm/s ≤ *P_app_* < 1 × 10^−6^ cm/s are classified as having mediocre permeability, and compounds found to have high permeability have a *P_app_* ≥ 1 × 10^−6^ cm/s [33]. Compounds whose *P_app_* in the BBB model is <2.0 × 10^−6^ cm/s are known as poorly permeable. Compounds with questionable permeability have *P_app_* values in the range of 2.0 to 4.0 × 10^−6^ cm/s. Substances that have a *P_app_* value greater than 4.0 × 10^−6^ cm/s are regarded as highly permeable [34].

### 2.11. Analysis of the Results

For the statistical analysis, Statistica 13.3 software (StatSoft Poland, Krakow, Poland) was employed. Data are presented as mean values ± standard deviations. Experimental data were analyzed using the skewness and kurtosis tests to determine the normality of each distribution, and Levene’s test assessed the equality of variances. To establish statistical significance, a one-way analysis of variance (ANOVA) was carried out, followed by the Bonferroni post hoc test, which facilitated the comparison of experimental outcomes for each extract. Noteworthy differences were considered significant at *p* < 0.05. Principal component analysis (PCA), which was carried out to explain and interpret interdependence between the compounds’ profile and their impact on the extracts’ biological activity was performed using PQStat v.1.8.4.140 software (Poznań, Poland). The Pearson matrix was also calculated with PQStat v.1.8.4.140 software. To determine the extract with the strongest potential neuroprotective activity, which will include both antioxidant activity (DPPH, ABTS, CUPRAC, FRAP methods) and the ability to inhibit AChE, BChE, and tyrosinase enzymes, a multidimensional comparative analysis (MCA), which compares multi-feature objects, was performed [35,36]. Synthetic indicators are the main criterion for organizing the examined results and their ranking, with the use of multidimensional comparative analysis. During the analysis, destimulants were converted into stimulants. In the process of normalization, the considered diagnostic features have been assigned a specific meaning for the assessment of objects. Standardization was used for the normalization of variables. Synthetic measures were calculated, and rankings of regions were prepared.

## 3. Results

The research began with the preparation of the scCO_2_ extracts (LFE-CO_2_-1–6) as a result of using the process conditions pressure 2000–5000 PSI and 40–50 °C and ethanol extracts of post-scCO_2_ plant material (rLFE-EtOH-1–6). Subsequently, chromatographic analysis to determine the contents of active constituents such as xanthohumol and lupulone content (Table 3) was performed. Xanthohumol is a prenylated chalcone, whilst lupulone is a β-acid and they differ in lipophilicity. These compounds, empowered by other ingredients present in the hop strobile extract which might act synergistically, have antioxidant and neuroprotective properties [37,38]. Thus, the determination of the biological activity of extracts in this direction was carried out.

scCO_2_ enabled the extraction of high amounts of lupulone; however, the content of xanthohumol in these extracts was low, as the highest obtained content was found in LFE-CO_2_-6 (0.304 ± 0.005 mg xanthohumol/g plant material). The highest content of lupulone was extracted at 3500 PSI (14.629 ± 0.250 mg lupulone/g plant material) and 5000 PSI (14.432 ± 0.184 mg lupulone/g plant material) at 50 °C. Extraction of the plant material remaining in the vessel after scCO_2_ extraction allowed for the obtaining of higher amounts of xanthohumol, and the highest content was noted in rLFE-EtOH-4: 1.732 ± 0.016 mg xanthohumol/g plant material.

Total phenolic content (TPC) was also analyzed to define the profile of secondary plant metabolites present in extracts (Table 4). In the scCO_2_ extracts the TPC content increased with the temperature and pressure of the process; at 40 °C and 2000 PSI, 22.765 ± 1.179 mg GAE/g plant material was obtained and at 50 °C and 5000 PSI, 38.263 ± 0.965 mg GAE/g plant material was obtained. However, after scCO_2_ extraction, the remaining plant material was still rich in phenolic compounds, as the TPC was assessed to be from 29.981 ± 0.977 mg GAE/g plant material in rLFE-EtOH-5 to 38.782 ± 1.706 mg GAE/g plant material in rLFE-EtOH-2.

After determining the content of active biological compounds in the extracts, their neuroprotective potential was studied. There are several enzymes whose inhibition may help prevent neurodegeneration; among them are AChE and BChE. These two enzymes break down the neurotransmitter acetylcholine, which is essential for proper cognitive function [39]. Subsequently, this leads to an increase in acetylcholine levels in the brain, which can improve cognitive function and delay the progression of neurodegenerative disorders [40]. Last but not least, inhibition of tyrosinase, which is not considered a direct target for the prevention of neurodegeneration, might be helpful in neuroprotection. This enzyme converts tyrosine to L-DOPA and then to dopaquinone [41]. By inhibiting the activity of tyrosinase, the degradation of L-DOPA is reduced, resulting in higher levels of dopamine precursors. This is particularly relevant for Parkinson’s disease, as the condition is characterized by a deficiency of dopamine [42]. In Parkinson’s disease, there is selective degeneration of neuromelanin-containing neurons, especially substantia nigra dopaminergic neurons [43]. Neuromelanin levels increase as humans age, which is a major risk factor for developing Parkinson’s disease. However, the role of neuromelanin in Parkinson’s disease is not well-understood yet. Therefore, inhibiting tyrosinase can help to increase dopamine levels and improve symptoms.

The potential of extracts to inhibit AChE, BChE, and tyrosinase was expressed as IC_50_ (Table 5), and thus a smaller value means better potential for inhibiting the enzyme. It can be noticed that the higher the pressure of the extraction process, the stronger the inhibitory activity of enzymes. In all of the assays, the most promising inhibiting potential was noticed for LFE-CO_2_-6—IC_50_ values 9.759 ± 0.433 mg/mL, 2.661 ± 0.021 mg/mL, and 2.821 ± 0.187 in AChE, BChE, and tyrosinase, respectively. The post-scCO_2_ ethanol group showed worse results than the scCO_2_ extracts (Supplementary Materials, Table S1), and the most inhibitory potential was noted for rLFE-EtOH-4, with IC_50_ 80.071 ± 1.002 mg/mL, 39.063 ± 0.387 mg/mL, and 23.348 ± 0.817 mg/mL in AChE, BChE, and tyrosinase, respectively. The results were not greater than those for the reference, namely galantamine, whose IC_50_ is 19.112 ± 0.871 μg/mL in AChE and 161.389 ± 7.183 μg/mL in BChE. However, in the tyrosinase inhibition assay, the result obtained for LFE-CO_2_-6 was greater than the reference, azelaic acid, whose IC_50_ is 6.455 ± 0.249 mg/mL.

Neurodegenerative diseases are often associated with increased oxidative stress and inflammation in the brain, which can cause damage to neurons and contribute to their dysfunction and death. Antioxidants work by neutralizing free radicals, supporting neuroprotective effects. The potential of extracts to scavenge radicals was studied in the ABTS and DPPH assay, whilst the ability to inhibit oxidation reactions by being oxidized themselves was studied using the FRAP and CUPRAC methods (Table 6 and Table 7). The greatest results in scCO_2_ extracts were obtained for higher temperatures and, more noticeably, at higher pressure (Table 6). In ABTS studies, the greatest result was obtained for LFE-CO_2_-6—29.504 ± 1.038 mg trolox/g plant material, whilst in DPPH measurements the greatest potential was noticed in LFE-CO_2_-5 (38.175 ± 0.198 mg trolox/g plant material), which was statistically similar to the antioxidant potential of LFE-CO_2_-6 (37.919 ± 0.176 mg trolox/g plant material). In both assays determining the reducing potential of the extract, LFE-CO_2_-6 was found to be the most potent (7.501 ± 0.242 mg trolox/g plant material) in the FRAP, and 47.996 ± 1.134 mg trolox/g plant material in the CUPRAC assay.

Similarly to TPC, the antioxidant potential was also noticed in the remaining plant material after scCO_2_ (Table 7). Interestingly, the antioxidant properties were stronger within this group than in the scCO_2_ group, as in ABTS the highest result was 49.424 ± 2.094 mg trolox/g plant material in rLFE-EtOH-4 and in DPPH (41.450 ± 1.286 mg trolox/g plant material) for rLFE-EtOH-2. The reducing potential was seen to be the strongest in rLFE-EtOH-4 (15.436 ± 0.752 mg trolox/g plant material) in FRAP, whilst in CUPRAC it was in rLFE-EtOH-2 (61.096 ± 1.438 mg trolox/g plant material).

Transition metals induce the formation of free radicals, which results in increased oxidative stress and the progression of neurodegenerative changes. In the therapy and prevention of these diseases, metal chelation is very important, because it is possible to reduce the level of free radicals that act destructively on many cellular structures, including proteins, lipids, and nucleic acids. The following assay determined the extracts’ ability to chelate Fe^2+^ ions. During the assay, Fe^2+^ ions that were not bound by the extracts were combined with ferrozine to form a colored complex. The color intensity decreased with the increasing chelating capacity of the extracts, and was measured spectrophotometrically [44].

**Figure 2:**
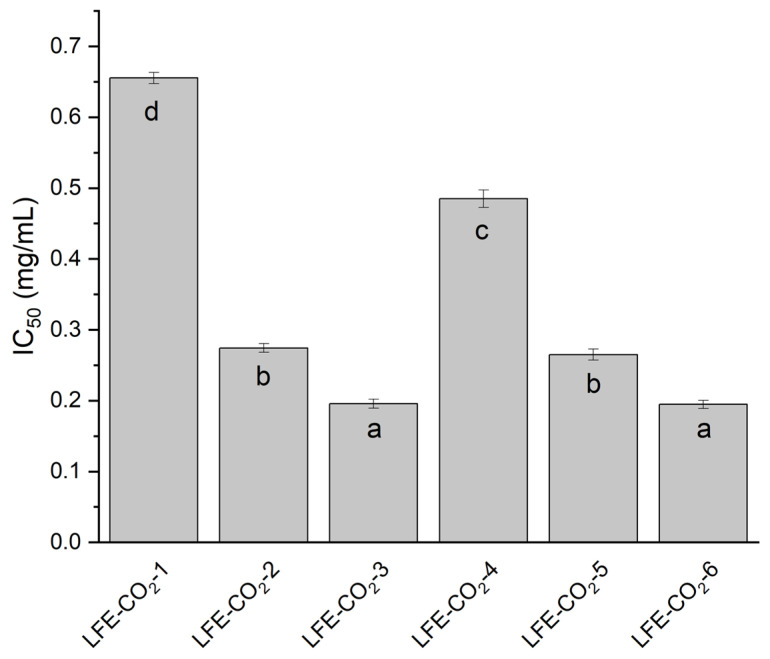
The chelating activity of CO_2_ hop strobile extracts presented as IC_50_ (mg/mL). Different letters (a–d) within the bars indicate statistical difference (*p* < 0.05).

The chelating ability was presented as IC_50_, and thus, the smaller the result, the greater the potential of the extract (Figure 2). At least twice-better results were obtained in the scCO_2_ group than in the post-scCO_2_ ethanol group (Supplementary Materials, Table S2). The higher the temperature, and, more importantly, the higher the pressure used during extraction, the better the chelating properties of the extract. The most outstanding results were noticed for LFE-CO_2_-6 (0.195 ± 0.006 mg/mL) and for LFE-CO_2_-3 (0.196 ± 0.006 mg/mL), which were statistically better than other extracts obtained under lower pressure. The results obtained for ethanol extracts of the remaining plant material after scCO_2_ extraction were noticeably worse than for scCO_2_. Among the post-scCO_2_ ethanol group, the best result was 0.887 ± 0.011 mg/mL for rLFE-EtOH-4. Nevertheless, all of the results were greater than for a reference substance, quercetin, whose IC_50_ is 1.46 mg/mL (calculated as prepared concentration) [30].

**Figure 3:**
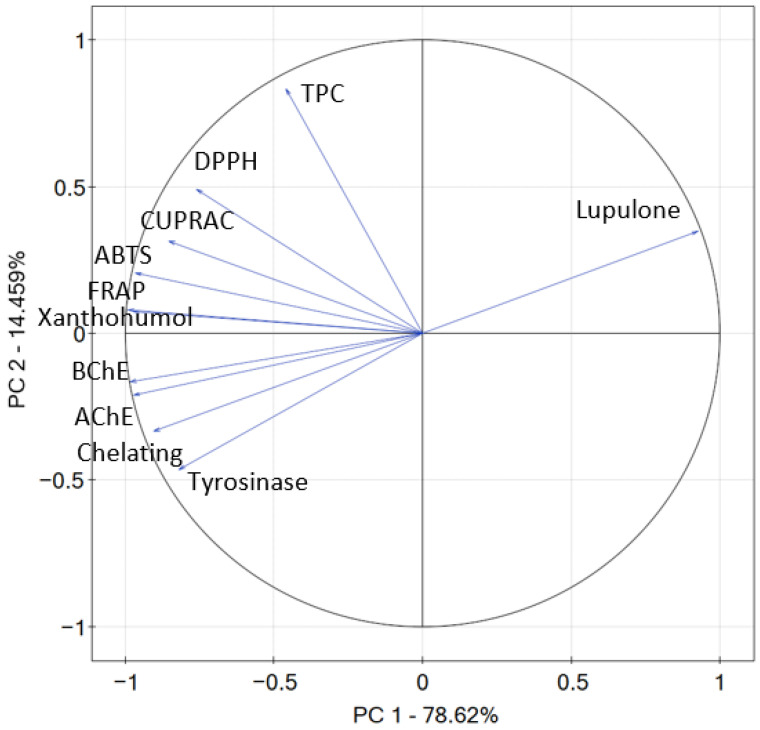
Contributions of variables (xanthohumol, lupulone content, TPC, ABTS, DPPH, FRAP, CUPRAC, chelating, AChE, BChE, tyrosinase) to PCs.

To describe the dependencies between studied plant material, the content of secondary plant metabolites, and the biological activity of the extracts, PCA was performed. A total of 78.62% of the variation in the samples was explained by factor 1 (principal component PC1) and 14.59% was expressed by factor 2 (principal component PC2) (Figure 3). Lupulone content is the only parameter that correlates positively with PC1; all of the other parameters correlate negatively with PC1. Antioxidant potential, the contents of xanthohumol and lupulone, and TPC correlate positively with PC2, whilst inhibition of the enzymes and chelating activity correlate negatively with PC2. The potential of the extracts to inhibit AChE, BChE, tyrosinase, and chelating activity is strongly positively correlated. However, these parameters are strongly negatively correlated with lupulone content (−0.978, −0.977, −0.902, and −0.964, respectively), as the inhibitory potential was presented as IC_50_ values [45]. The antioxidant potential in ABTS, DPPH, FRAP, and CUPRAC assays of the extracts is positively correlated with the content of xanthohumol (0.984, 0.753, 0.986, and 0.813, respectively) and presents a good correlation in general with TPC, which is confirmed in the literature for various plant materials [46,47,48]. The biological activity observed in plant extracts is attributed to the interactions between different secondary plant metabolites [49,50,51]. These compounds work together to enhance their individual effects, leading to a more potent and diverse biological response. The combined action of these metabolites can result in increased efficacy and a broader spectrum of activity compared to isolated compounds.

Multidimensional comparative analysis is a valuable method for assessing and comparing extracts based on their diverse properties. This approach considers multiple factors, such as the ability to inhibit enzymes AChE, BChE, and tyrosinase, and the antioxidant activity studied in ABTS, DPPH, FRAP, and CUPRAC assays. By analyzing these aspects, the extract with the greatest neuroprotective potential was identified, with a crucial factor being the inhibition of enzymes involved in the development of neurodegeneration. In the overall ranking, the LFE-CO_2_-6 was determined as the one with substantial neuroprotective potential. Thus, LFE-CO_2_-6 and ethanolic extract of post-scCO_2_ plant material were chosen as a whole extract (LFE-CO_2_/EtOH) for complementing each other in biological activity and for complementing the content of compounds with biological activity; for this whole extract, four delivery systems of active compounds were prepared.

The systems of LFE-CO_2_/EtOH with Neusilin US2 and HP-β-CD were prepared (LFS 1–4) by freeze-drying to increase the solubility and permeability of active compounds in the extract. The systems were studied chromatographically, to assess the contents of xanthohumol and lupulone (Supplementary Materials, Table S3). The highest contents of xanthohumol and lupulone were noted for LFS-3, 64.406 ± 2.071 μg/100 mg of the system, as well as 385.269 ± 6.239 μg/100 mg of the system. Later, four systems were enrolled in an apparent solubility study in three media—FaSSGF, FaSSIF, and FeSSIF. Two compounds’ content and released % were studied: xanthohumol and lupulone.

**Figure 4:**
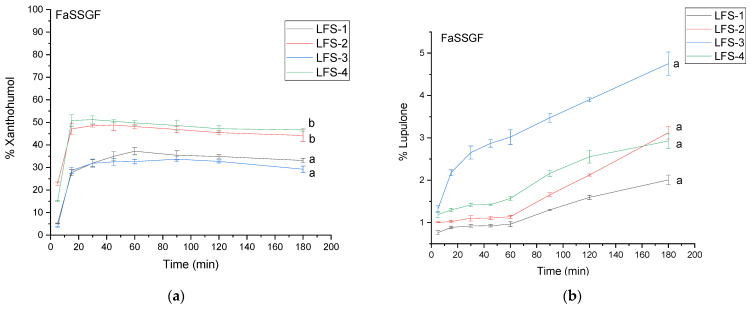
The dissolution profile of xanthohumol (**a**) and lupulone (**b**) in *Lupuli flos* systems (LFS) 1–4 in FaSSGF. Profiles with different letters differ significantly.

Xanthohumol is more soluble in acidic conditions than lupulone (Figure 4), reaching a maximum dissolution rate of 51.20 ± 1.59% in 30 min in LFS-4; however, xanthohumol undergoes degradation in acidic conditions as the profiles decrease after the first 30 min of the study. The literature confirms that prenylflavanoids are unstable at acidic pH [52]. LFS-3, and LFS-4 provided significantly better dissolution profiles than systems LFS-1 and LFS-2.

**Figure 5:**
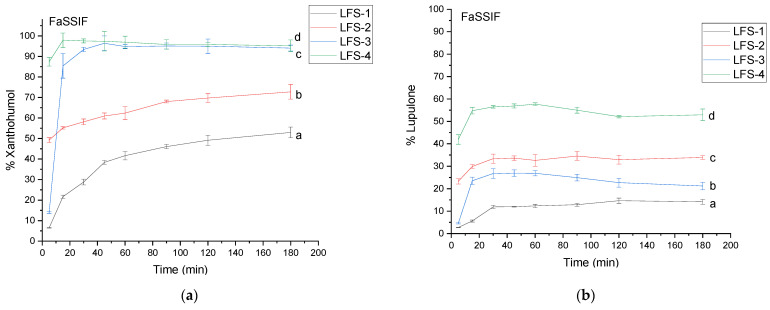
The dissolution profile of xanthohumol (**a**) and lupulone (**b**) in LFS 1–4 in FaSSIF. Profiles with different letters differ significantly.

Both xanthohumol and lupulone provide greater dissolution in intestinal conditions than in a gastric environment (Figure 5). However, xanthohumol is approx. two-times more soluble in fasted intestinal conditions than lupulone. LFS-4 provided an immediate release of xanthohumol (87.42 ± 2.09%), which was released within 5 min of the study, whilst lupulone reached the highest dissolution rate in the same system, LFS-4, reaching the level of 54–57% within 15 min of the study.

**Figure 6:**
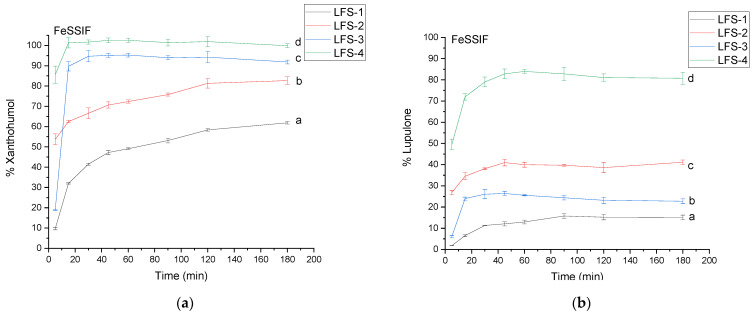
The dissolution profile of xanthohumol (**a**) and lupulone (**b**) in LFS 1–4 in FeSSIF. Profiles with different letters differ significantly.

Xanthohumol in fed intestinal conditions is slightly more soluble than in fasted conditions, similarily to lupulone (Figure 6). However, lupulone in LFS-4 in fed conditions dissolved significantly better, with 79.01 ± 2.29% released in 30 min. It might be beneficial to intake *Lupuli flos* systems after a meal, as it might lead to better bioavailability of secondary plant metabolite; however, in vivo studies are required to confirm this hypothesis. It is well-known that xanthohumol and lupulone are poorly soluble, with surfactants present in the media imitating the conditions present in the gastrointestinal tract that contribute to the wetting of the compounds [53].

Xanthohumol permeability in acidic conditions (Table 8) was described as poor *P_app_* 9.35 × 10^−7^ ± 1.29 × 10^−7^ cm/s; however, the results were greater than pH 6.8 (Table 9)–*P_app_* 7.30 × 10^−7^ ± 1.02 × 10^−7^ cm/s, as xanthohumol is a weak acid [54], and in acidic conditions it is present in undissociated form, whilst at higher pH values it is present in a more dissociated form (as a weak acid it does not completely dissociate). Lupulone was found to be more permeable than xanthohumol, as at pH 1.2 the *P_app_* value was 2.16 × 10^−6^ ± 4.61 × 10^−7^ cm/s and at pH 6.8 it was 2.79 × 10^−6^ ± 6.14 × 10^−8^ cm/s. The greatest improvement in xanthohumol’s permeability was noted at pH 1.2 for LFS-3 (*P_app_* 3.55 × 10^−6^ ± 3.80 × 10^−7^ cm/s); at pH 6.8 it was *P_app_* 2.50 × 10^−6^ ± 3.28 × 10^−7^ cm/s (LFS-4). In both cases, xanthohumol’s increase in permeability allowed us to define it as good. Lupulone permeability in pH 1.2 was elevated significantly in LFS-3, with *P_app_* value 4.28 × 10^−5^ ± 6.67 × 10^−7^ cm/s, and at pH 6.8 (5.55 × 10^−6^ ± 5.95 × 10^−7^ cm/s), and also for LFS-3.

The antioxidant potential of LFE-CO_2_/EtOH and four systems were also studied in ABTS, DPPH, FFRAP, and CUPRAC assays (Supplementary Materials, Table S4). The potential of LFE-CO_2_/EtOH in all methods was higher than for scCO_2_ and ethanol extract of post-scCO_2_ material separately, while the carriers themselves did not show antioxidant potential. The antioxidant activity remained statistically the same or slightly better in prepared systems.

## 4. Discussion

Hop strobiles and their constituents provide a variety of biological activities such as sedative [55], neuroprotective [56,57,58], anti-inflammatory [59], antimicrobial [60], antioxidant [61], antidiabetic [62], anticancer [63], and antimicrobial [64]. This study focused on *Lupuli flos* neuroprotective activity due to the fact that the statistics and forecasts for present and future neurodegenerative patients are not optimistic. The number of patients with dementia is rising, with nearly 10 million new cases each year [65].

The physicochemical properties of *Lupuli flos* extract constituents lead to poor bioavailability, and thus this study aimed, among other things, to obtain the extract with the greatest neuroprotective potential which will be involved in delivery systems that could overcome this limitation.

The research design presented in this publication allows the use of two different extraction techniques in order to obtain optimally high contents of lupulone and xanthohumol, which are responsible for the neuroprotective effect of the raw hop material. scCO_2_ extraction is known to be a green method, with a neutral impact on the environment, which efficiently extracts lipophilic bioactive compounds [66]. Thus, within this study, a higher content of β-acid, lupulone was obtained in scCO_2_ (medium content for all scCO_2_ extracts: 12.3 mg/g) than in ethanol extraction of the remaining plant material after scCO_2_ extraction (medium content: 1.5 mg/g). However, the content of xanthohumol provided by scCO_2_ extraction was low (mean value 0.14 mg/g), and thus additional ultrasound-assisted extraction was performed to determine whether more phenolic content could be obtained. According to Kowalczyk et al. [67], among water, methanol, and ethanol, the greatest polyphenolic and flavonoid content is obtained with ethanol; thus, within this study, ethanol was used as a solvent to extract plant material after scCO_2_ [67]. Additional extraction of post-scCO_2_ plant material resulted in extracts with a higher content of xanthohumol (mean value 1.4 mg/g).

Total phenolic content obtained with scCO_2_. (mean value 31.5 mg/g) was lower than the content obtained by ethanol post-scCO_2_. (35.5 mg/g). However, the results might be seen as quite similar. It might be a result of the fact that phenolic compounds, depending on the type, differ in lipophilicity. The TPC obtained within the study was comparable with other extraction methods and various cultivars [37]. In a Lyu et al. study [37], various hop cultivars were studied as water and hydroalcoholic extracts. The cultivars varied in total phenolic content, but it also depended on the solvent, as greater results were obtained in hydroalcoholic hop extracts (mean value 71.53 mg GAE g^−1^) than in aqueous extracts (mean value 57.88 mg GAE g^−1^), which was also noted in a Kowalczyk et al. study [67]. Santarelli et al. [68] studied extracts obtained by dynamic maceration, with the use of an ultrasound system high-power ultrasonic bath, and a high-pressure industrial process, obtaining between 34.2 and 48.5 mg GAE g^−1^. Similarly to the current study, higher temperatures provided higher polyphenolic content. In general, depending on the cultivar, temperature, pressure, and technique of extraction, various phenolic contents might be obtained. As Santarelli et al. [68] noted, the increase in TPC by elevating the temperature of extraction is attributed to various factors, including the breakdown of bonds between phenols and insoluble plant cell polymers (such as proteins and polysaccharides), leading to the release of free phenols. Additionally, higher temperatures can increase the solubility of phenolic compounds and improve mass transfer rates, ultimately increasing extraction efficiency.

The various secondary plant metabolite profiles in extracts lead to different biological potentials. Kobus-Cisowska et al. studied the cultivars Magnum, Lubelski, and Marynka of hop strobile, where a strong inhibition of AChE and BChE was provided for aqueous variants [69]. In another study, hop cones and leaves were studied as components of functional teas [70]. In combination with white mulberry, mint, and yellow tea leaves, hop cones showed high AChE inhibition, whilst BChE was strongly inhibited by a mixture containing hop leaves. Xanthohumol is determined as a strong cholinesterase inhibitor [71]. In an Orhan et al. study [72], AChE and BChE IC_50_ values of xanthohumol were provided as 71.34 μM, and 32.67 μM, respectively. In another study, the K_I_ values of xanthohumol were determined as 95.5 nM for AChE and 124.9 nM for BChE [73].

In other studies, xanthohumol inhibited both monophenolase and diphenolase activities of mushroom tyrosinase, with IC_50_ values of 15.4 mM and 31.1 mM, respectively [74]. The inhibition was evaluated using either L-tyrosine as a substrate for monophenolase activity or L-DOPA for diphenolase activity. Additionally, in normal human melanocytes from darkly pigmented skin cells, xanthohumol displayed a potent suppression of cellular tyrosinase activity [75]. Notably, this effect was observed at concentrations of 2.5–5 μM (36.57% and 47.61% of tyrosinase were inhibited at 2.5 and 5 μM, respectively), which are within the non-toxic range. Remarkably, the suppression of tyrosinase activity by xanthohumol did not coincide with any discernible impact on melanin synthesis within the cells.

Antioxidant activity is usually related to phenolic content in the extract [76]. The antioxidant potential studied in FRAP and DPPH assays was higher than in methanol, ethanol, and isopropanol extracts of hop leaves [77], as a consequence of higher TPC content; however, all of the obtained results were poorer than in the current study. In a study of young shoots of *Humulus lupulus*, DPPH activity was lower than in the current study (0.3–0.5 mg Trolox equivalents/g) [78]. The potential of currently studied extracts to scavenge the DPPH radical was greater than n-hexane cone extract (14.95 ± 0.03 µg Trolox equivalent/g) [79]. However, the antioxidant potential studied by Lyu et al. in DPPH and ABTS assays was stronger than in the current study [37]. The comparison of the results with the literature data might be challenging, as different authors use various concentrations of reagents, present the results as prepared concentration and as a final concentration, use various ratios of reagents, and present results in different ways (as equivalents, as IC_50_, and as a percentage of inhibition at a certain concentration), which makes comparison difficult and calls for a unification of the methods.

To the best of the authors’ knowledge, there is only one study on preparing powder systems of hop strobile extracts [80], which showed influence on the moisture content, water activity, color, flowability, microstructure, and water sorption behavior of the microencapsulates, but not the solubility. However, there are a few studies on improving the physicochemical properties of xanthohumol and isoxanthohumol, and these will be further referred to. By incorporating carriers with solubilizing properties (HP-β-CD and Neusilin US2), the researchers effectively overcome the challenge of poor solubility. This innovative approach not only improves the dissolution profiles of the extracts, but also enhances their apparent permeability coefficients, as demonstrated in the PAMPA model. To the best of the authors’ knowledge, dissolution profiles of lupulone have not been discussed in the literature before.

In a Sanchez-Aguinagalde et al. study, amorphous solid dispersions of xanthohumol in Poly(ε-caprolactone) were prepared in a solubility study performed in 4 mL 0.1 M PBS (pH 7.4) at 37 °C, where 2, 5 and 10 wt% XH blends released about 50, 150 and 300 ug in 1 h, 3 h and 4.5 h, respectively [81]. In a Budziak et al. study [19], xanthohumol cocrystal with acetamide resulted in a 2.6-fold enhancement in solubility in a study performed in 5 mL of 50:50 (*v*/*v*) ethanol–water mixture. Unfortunately, it is hard to compare our results to both of these studies, as the parameters of the assay are too different. In a Ronka et al. study [17], another hop strobile constituent, isoxanthohumol, underwent a solubility study in dialysis method in PBS and at a temperature of 37 °C with a 20% addition of ethanol. Polymeric micelles based on Pluronics P123 and F127 were not able to enhance isoxanthohumol water solubility effectively, as only 13% of the flavonoid was released after 120 h, which was similar to the 14% value obtained for crude isoxanthohumol. The Hanmantrao et al. [20] study demonstrated that loading xanthohumol into a liquid self-nanoemulsifying drug-delivery system resulted in a significant enhancement in drug dissolution (more than 75% of the xanthohumol was released in the first hour), while the use of a polysaccharide-based solid self-nanoemulsifying drug-delivery system restricted drug release in the upper GIT but allowed for rapid release in the presence of live fecal microbiota, indicating its potential as a microbial-triggered colon-targeted delivery system.

FaSSGF, FaSSIF, and FeSSIF are complex solutions designed to imitate human gastric and small-intestine conditions in fasted and fed states. They contain natural surfactants, providing more accurate simulations than conventional dissolution media for in vitro studies. In the current study on powder systems of hop strobile extract in gastric conditions, 51.20% of xanthohumol was released in 30 min in gastric conditions in the fasted state, while in intestinal conditions in fasted and fed state the dissolution reached 97.9% within 15 min, and 100% within 15 min, respectively.

Not only is xanthohumol poorly soluble, but it is also weakly permeable in the gastrointestinal tract [82]. Unfortunately, xanthohumol’s poor solubility, in combination with insufficient permeability, leads to poor bioavailability [83]. Computational studies were carried out to predict XN pharmacokinetic parameters and compared the results to anastrozole [84]. The Brain Or IntestinaL EstimateD permeation method (BOILED-egg) plot between lipophilicity and polarity showed that anastrozole permeates the blood–brain barrier, whilst xanthohumol shows passive absorption by the gastrointestinal tract. Research carried out on Caco-2 cells revealed that xanthohumol has a tendency to accumulate within the cytosol, binds to the cellular proteins [85], and has the capability to insert itself inside the lipid bilayer of cell membranes, making the rapid transmembrane transfer challenging. Facilitated transport was not responsible for the uptake of xanthohumol; instead, accumulation inside the Caco-2 cells was apparently the result of specific binding to cytosolic proteins [85]. The apparent permeability coefficient confirmed the poor permeability of xanthohumol, as in the AP-to-BL (apical-to-basolateral transport) direction it was assessed as 1.33 × 10^−7^ cm/s, and in the BL-to-AP direction (2.37 × 10^−7^ cm/s) [85]. In a study on solid lipid nanoparticles, in the Caco-2 model, xanthohumol permeation was increased 4.62-fold (A-B), and excretion was decreased 0.31-fold (B-A) [83]. In vivo studies showed that after oral administration of xanthohumol in rats, more than 80% of the products of the intestinal degradation were excreted in feces; only a small fraction of the ingested xanthohumol was absorbed, and the majority was excreted. Avula et al. showed, in a study on rats, that xanthohumol absorption is generally poor, no matter whether the animals were treated with xanthohumol or hops extract, and thus the hop extract did not have an influence on the oral bioavailability [86]. Legette et al. provided results from people showing that xanthohumol has a biphasic absorption pattern [87]. Plasma xanthohumol levels in people significantly increase only after a dose higher than 60 mg [87]. Our studies confirmed xanthohumol’s poor permeability in the PAMPA gastrointestinal model, as *P_app_* values were obtained for pure xanthohumol in the LFE-CO_2_/EtOH at pH 1.2 (9.35 × 10^−7^ ± 1.29 × 10^−7^ (cm/s)) and 6.8 (7.30 × 10^−7^ ± 1.02 × 10^−7^ (cm/s)). However, the *P_app_* was increased for the systems to 3.55 × 10^−6^ ± 3.80 × 10^−7^ cm/s (LFS-3) at pH 1.2, and 2.50 × 10^−6^ ± 3.28 × 10^−7^ (LFS-4) at pH 6.8, with the values described as highly permeable.

*Lupuli flos* contains α-acids (humulone, cohumulone, and adhumulone), and β-acids (lupulone, colupulone, and adlupulone). Cattoor et al. studied the GIT permeability of α-acids and β-acids across Caco-2 monolayers [88]. The permeability coefficients for apical-to-basolateral transport for α-acids were 14 × 10^−6^ to 41 × 10^−6^ cm/s, whilst for β-acids the results were worse (0.9 × 10^−6^ to 2.1 × 10^−6^ cm/s), and thus β-acid permeability is low, and limiting factors in the absorption of β-acids could involve P-gp and MRP-2-type efflux transporters and phase II metabolism [88]. Our results are similar to the ones obtained in Caco-2 cell lines, as *P_app_* values for lupulone were 2.16 × 10^−6^ ± 4.61 × 10^−7^ at pH 1.2 and 2.79 × 10^−6^ ± 6.14 × 10^−8^ at pH 6.8. The values were improved after introducing the extract into the systems to 4.28 × 10^−5^ ± 6.67 × 10^−7^ (LFS-3) at pH 1.2, and to 5.55 × 10^−6^ ± 5.95 × 10^−7^ (LFS-3) at pH 6.8.

*Lupuli flos* constituents were proved to cross the blood–brain barrier; e.g., in a study by Sekiguchi et al., 6-prenylnaringenin permeated the BBB in mice [9], and it was also suggested that 8-prenylnaringenin could penetrate the blood–brain barrier in vivo study [11]. The xanthohumol ability to permeate the BBB was confirmed in mice, where the compound was able to cross the blood–brain barrier in quantifiable amounts [8]. PAMPA BBB confirmed that both xanthohumol, and lupulone can cross the blood–brain barrier (7.18 × 10^−7^ ± 1.92 × 10^−7^ cm/s, and 5.35 × 10^−6^ ± 5.18 × 10^−7^ cm/s, respectively).

The research not only highlights the potential neuroprotective effects of hop strobile extracts, but also introduces a strategic approach to overcoming solubility limitations and enhancing permeability, thereby elevating their overall efficacy after oral administration. The use of carriers allowed for the preservation of the biological activity of the extracts, holding promise for the development of neurodegeneration prevention.

## 5. Conclusions

scCO_2_ extraction is an efficient technique for obtaining extracts of *Lupuli flos* with neuroprotective potential. However, in the case of extracts of *Lupuli flos*, it is valuable to combine it with ethanolic extraction to withdraw phenolic compounds which are responsible for antioxidant activity, combined with a neuroprotective profile. Delivery systems of xanthohumol and lupulone based on connections with magnesium aluminometasilicate and hydroxypropyl-β-cyclodextrin provided high dissolution rates in both gastric and intestinal environments, whether in a fasted or fed state. In addition, in the case of selected systems, it was possible to increase the permeability of xanthohumol and lupulone through the GIT barrier as a consequence of improving the dissolution dynamics of those lipophilic compounds.

## Figures and Tables

**Table 1:** The parameters of supercritical carbon dioxide (scCO_2_) extraction and ultrasound-assisted extraction of hop strobiles. LFE—*Lupuli flos* extract, rLFE—extract from the remaining plant material after scCO_2_ extraction, EtOH—ethanol.

Name of the Extracts	Plant Material	Solvent	Temperature	Pressure
LFE-CO_2_-1	Dried plant material	scCO_2_	40 °C	2000 PSI
LFE-CO_2_-2			40 °C	3500 PSI
LFE-CO_2_-3			40 °C	5000 PSI
LFE-CO_2_-4			50 °C	2000 PSI
LFE-CO_2_-5			50 °C	3500 PSI
LFE-CO_2_-6			50 °C	5000 PSI
rLFE-EtOH-1	Plant material after scCO_2_ extraction	70% (*v*/*v*) EtOH	40 °C	-
rLFE-EtOH-2			40 °C	
rLFE-EtOH-3			40 °C	
rLFE-EtOH-4			50 °C	
rLFE-EtOH-5			50 °C	
rLFE-EtOH-6			50 °C	

**Table 2:** The composition of *Lupuli flos* extract systems. HP-β-CD—hydroxypropyl-β-cyclodextrin, LFS—*Lupuli flos* system.

Extract	Carrier 1	Carrier 2	Ratio	Name
LFE-CO_2_-6 + rLFE-EtOH-6 (LFE-CO_2_/EtOH)	Neusilin US2	-	1:1.5	LFS-1
LFE-CO_2_-6 + rLFE-EtOH-6 (LFE-CO_2_/EtOH)	Neusilin US2	HP-β-CD	1:1:0.5	LFS-2
LFE-CO_2_-6 + rLFE-EtOH-6 (LFE-CO_2_/EtOH)	HP-β-CD	-	1:1.5	LFS-3
LFE-CO_2_-6 + rLFE-EtOH-6 (LFE-CO_2_/EtOH)	HP-β-CD	Neusilin US2	1:1:0.5	LFS-4

**Table 3:** The content of xanthohumol and lupulone present in *Lupuli flos* extracts, presented as mg of the compound/g plant material. Means with different superscript letters (a–i) within the same column differ significantly (*p* < 0.05).

*Lupuli flos* scCO_2_/Ethanol Extracts	Xanthohumol	Lupulone
	Amount of Active Compounds (mg)/Dry Plant Material (g)	
LFE-CO_2_-1	0.050 ± 0.001 ^a^	10.225 ± 0.093 ^f^
LFE-CO_2_-2	0.115 ± 0.002 ^b^	12.693 ± 0.152 ^g^
LFE-CO_2_-3	0.165 ± 0.002 ^c^	12.671 ± 0.198 ^g^
LFE-CO_2_-4	0.062 ± 0.002 ^a^	8.860 ± 0.056 ^e^
LFE-CO_2_-5	0.113 ± 0.002 ^b^	14.629 ± 0.250 ^h^
LFE-CO_2_-6	0.304 ± 0.005 ^d^	14.432 ± 0.184 ^h^
rLFE-EtOH-1	1.250 ± 0.008 ^e^	1.701 ± 0.041 ^c^
rLFE-EtOH-2	1.405 ± 0.013 ^g^	0.889 ± 0.010 ^b^
rLFE-EtOH-3	1.317 ± 0.013 ^f^	0.737 ± 0.012 ^a,b^
rLFE-EtOH-4	1.732 ± 0.016 ^i^	4.924 ± 0.020 ^d^
rLFE-EtOH-5	1.449 ± 0.019 ^h^	0.579 ± 0.013 ^a,b^
rLFE-EtOH-6	1.234 ± 0.017 ^e^	0.249 ± 0.007 ^a^

**Table 4:** The content of polyphenols (TPC) in the studied hop strobile extracts was determined as mg gallic acid equivalents (GAE)/g plant material. Means with different superscript letters (a–e) differ significantly (*p* < 0.05).

*Lupuli flos* scCO_2_/Ethanol Extracts	Total Phenolic Content
	mg GAE/g
LFE-CO_2_-1	22.765 ± 1.179 ^a^
LFE-CO_2_-2	29.529 ± 0.959 ^b^
LFE-CO_2_-3	31.630 ± 1.791 ^b,c^
LFE-CO_2_-4	30.145 ± 0.542 ^b^
LFE-CO_2_-5	36.803 ± 1.538 ^c,d,e^
LFE-CO_2_-6	38.263 ± 0.965 ^d,e^
rLFE-EtOH-1	35.953 ± 1.773 ^c,d,e^
rLFE-EtOH-2	38.782 ± 1.706 ^d,e^
rLFE-EtOH-3	33.302 ± 2.038 ^b,c,d^
rLFE-EtOH-4	41.457 ± 1.162 ^e^
rLFE-EtOH-5	29.981 ± 0.977 ^b^
rLFE-EtOH-6	33.476 ± 1.179 ^b,c,d^

**Table 5:** Inhibition of acetylcholinesterase (AChE), butyrylcholinesterase (BChE), and tyrosinase by scCO_2_ hop strobile extracts presented as IC_50_ (mg/mL). Means with different superscript letters (a–f) within the same column differ significantly (*p* < 0.05).

*Lupuli flos* scCO_2_ Extracts	AChE	BChE	Tyrosinase
	IC_50_ (mg Plant Material/mL)		
LFE-CO_2_-1	25.775 ± 1.100 ^d^	7.280 ± 0.085 ^e^	21.537 ± 0.300 ^e^
LFE-CO_2_-2	16.696 ± 0.763 ^b^	3.760 ± 0.106 ^b^	16.997 ± 1.006 ^d^
LFE-CO_2_-3	12.476 ± 0.408 ^a^	4.023 ± 0.015 ^c^	7.760 ± 0.359 ^b^
LFE-CO_2_-4	32.015 ± 0.708 ^e^	10.934 ± 0.065 ^f^	17.963 ± 0.246 ^d^
LFE-CO_2_-5	20.299 ± 1.520 ^c^	4.321 ± 0.037 ^d^	13.764 ± 0.233 ^c^
LFE-CO_2_-6	9.759 ± 0.433 ^a^	2.661 ± 0.021 ^a^	2.821 ± 0.187 ^a^

**Table 6:** Antioxidant potential of CO_2_ *Lupuli flos* extracts presented as mg trolox/g plant material. Means with different superscript letters (a–d) within the same column differ significantly (*p* < 0.05).

*Lupuli flos* scCO_2_ Extracts	ABTS	DPPH	FRAP	CUPRAC
	mg Trolox/g Plant Material			
LFE-CO_2_-1	17.868 ± 0.069 ^a^	35.952 ± 0.074 ^b^	5.714 ± 0.069 ^a^	31.465 ± 1.522 ^a^
LFE-CO_2_-2	20.562 ± 0.941 ^a,b^	37.689 ± 0.085 ^c,d^	6.303 ± 0.260 ^a,b^	47.924 ± 1.363 ^c^
LFE-CO_2_-3	22.378 ± 0.879 ^b,c^	36.959 ± 0.204 ^c^	6.865 ± 0.360 ^b,c^	46.678 ± 1.076 ^c^
LFE-CO_2_-4	19.337 ± 0.260 ^a^	33.094 ± 0.350 ^a^	5.404 ± 0.292 ^a^	37.044 ± 0.478 ^b^
LFE-CO_2_-5	23.602 ± 0.933 ^c^	38.175 ± 0.198 ^d^	7.013 ± 0.291 ^b,c^	46.092 ± 1.023 ^c^
LFE-CO_2_-6	29.504 ± 1.038 ^d^	37.919± 0.176 ^d^	7.501 ± 0.242 ^c^	47.996 ± 1.134 ^c^

**Table 7:** Antioxidant potential of *Lupuli flos* extracts presented as mg trolox/g plant material. Means with different superscript letters (a–c) within the same column differ significantly (*p* < 0.05).

*Lupuli flos* Ethanol Extracts	ABTS	DPPH	FRAP	CUPRAC
	mg Trolox/g Plant Material			
rLFE-EtOH-1	41.710 ± 1.922 ^a^	40.214 ± 1.446 ^a^	12.964 ± 0.650 ^a^	51.787 ± 0.506 ^a^
rLFE-EtOH-2	45.640 ± 2.113 ^a^	41.450 ± 1.286 ^a^	15.020 ± 0.778 ^a^	61.096 ± 1.438 ^c^
rLFE-EtOH-3	41.591 ± 1.339 ^a^	37.603 ± 1.156 ^a^	14.121 ± 0.533 ^a^	57.259 ± 1.462 ^a,b,c^
rLFE-EtOH-4	49.424 ± 2.094 ^a^	41.439 ± 0.976 ^a^	15.436 ± 0.752 ^a^	53.765 ± 2.785 ^a,b^
rLFE-EtOH-5	42.425 ± 2.327 ^a^	38.345 ± 1.672 ^a^	14.137 ± 0.699 ^a^	56.134 ± 1.753 ^a,b,c^
rLFE-EtOH-6	42.875 ± 3.043 ^a^	40.973 ± 0.989 ^a^	14.935 ± 0.837 ^a^	59.197 ± 1.245 ^b,c^

**Table 8:** Gastrointestinal permeability of xanthohumol and lupulone in LFE-CO_2_/EtOH, and systems LFS 1–4 at pH 1.2 Columns with different superscript letters (a–d) differ significantly.

Extract/System	Xanthohumol	Lupulone
	*P_app_* (cm/s)	
LFE-CO_2_/EtOH	9.35 × 10^−7^ ± 1.29 × 10^−7 a^	2.16 × 10^−6^ ± 4.61 × 10^−7 a^
LFS-1	1.84 × 10^−6^ ± 1.36 × 10^−7 a,b^	8.83 × 10^−6^ ± 5.71 × 10^−7 b^
LFS-2	2.29 × 10^−6^ ± 1.99 × 10^−7 b^	1.28 × 10^−5^ ± 5.43 × 10^−7 b,c^
LFS-3	3.55 × 10^−6^ ± 3.80 × 10^−7 c^	4.28 × 10^−5^ ± 6.67 × 10^−7 d^
LFS-4	2.38 × 10^−6^ ± 3.58 × 10^−7 b^	1.60 × 10^−5^ ± 2.59 × 10^−6 c^

**Table 9:** Gastrointestinal permeability of xanthohumol and lupulone in LFE-CO_2_/EtOH, and systems LFS 1–4 at pH 6.8 Columns with different superscript letters (a, b) differ significantly.

Extract/System	Xanthohumol	Lupulone
	*P_app_* (cm/s)	
LFE-CO_2_/EtOH	7.30 × 10^−7^ ± 1.02 × 10^−7 a^	2.79 × 10^−6^ ± 6.14 × 10^−8 a^
LFS-1	1.07 × 10^−6^ ± 1.34 × 10^−7 a^	2.84 × 10^−6^ ± 4.21 × 10^−7 a^
LFS-2	1.26 × 10^−6^ ± 1.99 × 10^−7 a^	2.71 × 10^−6^ ± 8.69 × 10^−8 a^
LFS-3	2.07 × 10^−6^ ± 1.33 × 10^−7 b^	5.55 × 10^−6^ ± 5.95 × 10^−7 b^
LFS-4	2.50 × 10^−6^ ± 3.28 × 10^−7 b^	3.69 × 10^−6^ ± 5.38 × 10^−8 a^

## Data Availability

Data are available in a publicly accessible repository.

## Supplementary Materials

The following supporting information can be downloaded at: https://www.mdpi.com/article/10.3390/antiox12091722/s1, Table S1: Inhibition of acetylcholinesterase (AChE), butyrylcholinesterase (BChE), and tyrosinase by hop strobile extracts presented as IC_50_ (mg/mL), Table S2: Chelating activity of hop strobile extracts presented as IC_50_ (mg/mL), Table S3: The content of xanthohumol and lupulone in the prepared systems presented as μg of the compound/100 mg of the system, Table S4: Antioxidant activity of LFE-CO_2_/EtOH and the prepared systems presented as mg trolox/g plant material.

## Abbreviations

ABTS	2:2′-azino-bis(3-ethylbenzothiazoline-6-sulfonic acid)
AChE	Acetylcholinesterase
ANOVA	one-way analysis of variance
ATCI	acetylthiocholine iodide
BChE	butyrylcholinesterase
BTCI	butyrylthiocholine iodide
CUPRAC	cupric reducing antioxidant capacity
DAD	diode array detector
DPPH	2,2-diphenyl-1-picrylhydrazyl
DTNB	5,5′-dithio-bis-(2-nitrobenzoic) acid
EtOH	ethanol
FRAP	ferric reducing antioxidant power
HPLC	high-performance liquid chromatography
HP-β-CD	hydroxypropyl-β-cyclodextrin
LFE	*Lupuli flos* extract
LFS	*Lupuli flos* system
MCA	multidimensional comparative analysis
PC	principal component
PCA	principal component analysis
rLFE	remaining plant material after supercritical CO_2_ extraction
ROS	reactive oxygen species
scCO_2_	Supercritical carbon dioxide
TNB	3-carboxy-4-nitrothiolate
TPTZ	2,4,6-tris(2-pyridyl)-1,3,5-triazine
