# Supplementary material for: Hop Flower Supercritical Carbon Dioxide Extracts Coupled with Carriers with Solubilizing Properties—Antioxidant Activity and Neuroprotective Potential
Source: Antioxidants (Basel). 2023 Sep 5;12(9):1722. doi: 10.3390/antiox12091722 (PMC10525257; doi:10.3390/antiox12091722)
Supplement: Supplementary file 1 [file antioxidants-12-01722-s001.zip › antioxidants-2554599-supplementary.pdf]

Supplementary materials

Table S1. Inhibition of acetylcholinesterase (AChE), butyrylcholinesterase (BChE), and tyrosinase by hop strobile extracts presented as IC_{50} (mg/mL). Means with different superscript letters (a–d) within the same column differ significantly ($p < 0.05$). rLFE – extract from remaining plant material after supercritical CO_2 extraction, EtOH – ethanol

Extract	AChE	BChE	Tyrosinase
	IC_{50} (mg plant material/mL)		
rLFE-EtOH-1	85.956 ± 2.796^{a,b}	44.606 ± 0.999^{b,c}	28.049 ± 1.088^{b}
rLFE-EtOH-2	86.275 ± 0.884^{a,b}	46.528 ± 0.646^{b,c}	37.349 ± 0.882^{c}
rLFE-EtOH-3	93.974 ± 2.071^{c,d}	48.174 ± 0.534^{c}	50.747 ± 0.571^{d}
rLFE-EtOH-4	80.071 ± 1.002^{a}	39.063 ± 0.387^{a}	23.348 ± 0.817^{a}
rLFE-EtOH-5	88.417 ± 1.116^{b,c}	43.409 ± 1.109^{a,b}	37.478 ± 0.818^{c}
rLFE-EtOH-6	97.557 ± 1.630^{d}	47.446 ± 2.636^{b,c}	51.727 ± 0.542^{d}

Table S2. Chelating activity of hop strobile extracts presented as IC_{50} (mg/mL). Means with different superscript letters (a–d) within the same column differ significantly ($p < 0.05$). rLFE – extract from remaining plant material after supercritical CO_2 extraction, EtOH – ethanol

Extract	Chelating Fe^{2+} ions
	IC_{50} (mg/mL)
rLFE-EtOH-1	1.029 ± 0.030^{c,d}
rLFE-EtOH-2	1.102 ± 0.048^{d}
rLFE-EtOH-3	0.979 ± 0.053^{b,c}
rLFE-EtOH-4	0.872 ± 0.017^{a}
rLFE-EtOH-5	1.009 ± 0.038^{c,d}
rLFE-EtOH-6	0.852 ± 0.021^{a,b}

Table S3. The content of xanthohumol and lupulone in the prepared systems presented as µg of the compound/100 mg of the system. LFS – *Lupuli flos* system

System	Xanthohumol	Lupulone
	Content (µg /100 mg of the system)	
LFS-1	45.604 ± 1.211	189.438 ± 3.845
LFS-2	47.169 ± 1.618	190.003 ± 4.402
LFS-3	64.406 ± 2.071	385.269 ± 6.239
LFS-4	64.386 ± 1.788	321.655 ± 5.203

Table S4. Antioxidant activity of LFE-CO_2/EtOH and the prepared systems presented as mg trolox/g plant material. Means with different superscript letters (a, b) within the same column differ significantly ($p < 0.05$). LFE – *Lupuli flos* extract, EtOH – ethanol, LFS – *Lupuli flos* system

Extract	ABTS	DPPH	FRAP	CUPRAC
	mg trolox/g plant material			
LFE-CO_2/EtOH	53.391 ± 1.590^{a}	49.579 ± 1.108^{a,b}	22.181 ± 0.920^{a}	69.992 ± 1.749^{a}
LFS-1	53.657 ± 1.738^{a}	46.058 ± 1.200^{a}	23.539 ± 0.659^{a}	66.726 ± 1.577^{a}
LFS-2	55.121 ± 1.161^{a}	49.761 ± 0.526^{a,b}	23.270 ± 0.230^{a}	71.526 ± 1.250^{a}
LFS-3	56.654 ± 0.524^{a}	52.391 ± 1.414^{b}	25.020 ± 1.295^{a}	72.906 ± 1.217^{b}
LFS-4	56.317 ± 1.043^{a}	52.098 ± 1.803^{b}	24.356 ± 0.818^{a}	71.286 ± 1.422^{a}
